# Access to psychosocial support for church-going young people recovering from drug and substance abuse in Zimbabwe: a qualitative study

**DOI:** 10.1186/s12889-023-15633-8

**Published:** 2023-04-20

**Authors:** Faustina F. Muswerakuenda, Paddington T. Mundagowa, Clara Madziwa, Fadzai Mukora-Mutseyekwa

**Affiliations:** 1 College of Social Sciences, Theology and Education, Africa University, Mutare, Zimbabwe; 2 Clinical Research Center, Africa University, Mutare, Zimbabwe; 3 Department of Epidemiology and Biostatistics, University of South Carolina, Columbia, USA

**Keywords:** Substance abuse, Youths, Psycho-social support, Religion, Addiction

## Abstract

**Background:**

The church and other religious-affiliated organizations have promising yet underexplored potential to provide social support services for young people recovering from substance abuse in communities where drug and substance rehabilitation services are limited. This study aimed to establish the barriers and facilitators of accessing psychosocial support, the role of the church, and strategies to promote access to psychosocial support for youths recovering from drug and substance abuse.

**Methods:**

This was a qualitative cross-sectional study, and semi-structured interviews of 18 church-going youths and three youth pastors were conducted in eastern Zimbabwe. Data were collected using recorded telephone interviews. Data were transcribed and analyzed using the thematic network analysis technique of producing basic themes, which build into organizing themes. Organizing themes produces one overarching global theme. The Consolidated Criteria for Reporting Qualitative Research (COREQ) guidelines for reporting on qualitative research were used in reporting the study findings.

**Results:**

The interviews produced the following basic themes under organizing theme barriers: stigma and discrimination, parental/guardian denial, radical religious beliefs, and negative role models. Under the organizing theme facilitators, the basic themes were acceptance, confidentiality, peer and parental support, and an organized support program. The church acted as the bridge between the barriers to access to services and support seeking through innovative, inclusive projects and activities, as well as a pillar of social support.

**Conclusions:**

Acceptance of one’s addiction problem is critical to initiate seeking psychosocial support. Confidentiality, support from trustworthy relationships, and the availability of a well-coordinated recovery program enable young people to seek support. We recommend formal training church-based counselors in the ethical aspects of psychotherapy to reduce the preconceived social stigma associated with drug and substance abuse.

## Background

Globally, drug and substance abuse (DSA) is a leading cause of years lived with disability (YLDs) and was responsible for 183.9 million disability-adjusted life years (DALYs) [1]. The widespread and growing DSA epidemic has caused a devastating impact on adolescents and young people with an increased likelihood of developing substance use disorders (SUDs) [2, 3]. Substance use disorder is defined in this study as the recurrent use of alcohol and drugs that leads to clinically and functionally significant impairment [4]. Despite the high global burden of SUDs, there is a considerable treatment and support gap, particularly in low- and middle-income countries where between 76 and 85% of the people with the condition do not receive treatment [5]. The United Nations’ 2030 Sustainable Development Goals are pushing for the prioritization of action in improving mental health and substance use treatment and prevention [5, 6].

In addition to public and self-stigma due to negative stereotypes and prejudice [7], individuals who misuse alcohol and illicit substances are often marginalized and underserved by healthcare services due to provider-related stigma caused by the erroneous belief that addiction is caused by weak character and poor choices [8]. Thus, patients suffering from SUDs might be considered a lesser priority or less likely to be treated with dignity and compassion when compared with patients with other conditions. Though scarce in practice, interventions such as using social support services tailor-made for individuals who have committed to recovering from SUDs have been proven effective [9].

Besides the lack of facilities providing rehabilitation services for these individuals, most illicit drug users are unaware that they need support and treatment because they consider drugs a solution and not a problem [10]. Even after acknowledging that they are overwhelmed by the problem, most of them do not seek treatment because they: (i) may not be ready to stop, (ii) do not have health insurance, (iii) fear the effects of withdrawal symptoms on their job performance or (iv) do not know where to get treatment [11].

Zimbabwe adopted the World Health Organization (WHO) Special Initiative for Mental Health: Universal Coverage for Mental Health, launched in 2019 [12]. The aim was to address the SUDs and increase access to equitable mental health services. Drug and substance use in Zimbabwe has increased significantly during the past decade and is now rated number 8 in the country’s top ten risk factors accounting for DALYs [13]. Many young people take illicit alcohol, methamphetamine, codeine-containing cough syrups, marijuana/cannabis, and sedating opiates [14]. These illicit drugs are either clandestinely manufactured within the local communities or smuggled from neighboring countries through porous entry points and well-organized syndicates at the official border posts [15]. The drugs are then sold to the young people in many places where unlawful transactions take place sometimes by women who masquerade as innocent fruit vendors [16]. The DSA problem in Zimbabwe can be attributed to increased poverty and dwindling economic opportunities that have left many young people idle and depressed, only to find solace in illicit substance use. Similar to global reports, DSA is a significant challenge among Zimbabwean youths [17, 18]. According to the Zimbabwe Civil Liberties and Drugs Network (2019) report, the proportion of young people involved in DSA increased from 43% to 2017 to 57% in 2019 [19]. In 2019 about 45% of admitted mental health patients were youth abusing drugs [20].

As a result of the massive brain drain caused by the mass immigration of skilled mental health providers, there is a massive treatment gap in mental health and SUDs treatment in Zimbabwe [21]. There is a severe shortage of human resources for mental health, and the number of psychiatrists is estimated at 18 (0.1 per 100,000 population), 17 based in Harare [22]. Moreover, mental health services are chronically underfunded, with limited specialist treatment facilities [12]. Spiritual factors play a major role in mental health treatment in Zimbabwe. Many patients consult faith healers for managing psychosomatic and mental health disorders [23]. Spiritual powers, informal counseling, and rituals for healing are conducted as treatment by traditional and Christian faith healers [24].

### Role of religion in SDA

Religious institutions like churches serve as a proxy for information dissemination hubs and social connections and significantly influence individual behavior. The church and other religious-affiliated organizations have promising yet underexplored potential to provide social support services for treating SUDs in communities where substance use treatment services are limited [25]. The synchronicity of DSA among youths and the increase in churches within the local communities highlights the underutilized potential avenue to counter the scourge of substance use. However, stigma related to DSA is common among believers who are expected by society to live a venerated lifestyle. Thus, the substance abuser is confronted with the anticipated conflict between social stigma and the desire to live a reverent life. Anticipated stigma leads to failure to disclose one’s addiction problem and reduced likelihood of seeking treatment. The anticipated stigma associated with social rejection can further exacerbate the abuse leading to SUDs. The available scientific evidence shows that social support in the form of emotional comfort and knowledge about treatment options from significant others facilitate recovery from SUDs over time [26].

Practitioners often reserve their decision to discharge their clients because they feel that nonspecialized providers, such as self-help groups and religious organizations may be ill-prepared to address SUDs given their generalist training [27]. In addition, individuals providing these services in nonspecialty settings may have limited resources to effectively amplify treatment outcomes. Despite the controversy among addiction experts in clinical practice, research evidence reveals that partnering with faith-based organizations in the fight against DSA is a protective factor, particularly during recovery [28].

In Zimbabwe, some churches have been marred with abuse for political [29] and personal gains, as well as extortion and moral misconduct. Nevertheless, many religious organizations have been actively involved in mediating and ameliorating conflicts from an individual to national levels and established community projects such as social safety nets, education, and health facilities. Religious involvement can mitigate indulgence in illicit substance use and its adverse outcomes [28, 30]. To bridge the support and treatment gap in resources-limited contexts like Zimbabwe, we hypothesized that faith-based DSA recovery support and treatment could positively impact community prevention and treatment of substance use disorders and reduce relapses post-recovery. To assist church-going adolescents and young people recovering from DSA, the Africa University Clinical Research Centre established peer-support groups in which participants shared their experiences and solutions to the challenges they face. This study aimed to (i) establish the barriers and facilitators to accessing psychosocial support in a church context, (ii) determine the role of the church in DSA recovery, and (iii) explore strategies that the church can implement to promote access to psychosocial support to adolescents and young people recovering from DSA.

## Methods

### Study design

This was a cross-sectional study that employed qualitative methods and was conducted over seven weeks from January to March 2022. The researchers chose to use the qualitative research method because it highlights the process and delivers an in-depth understanding of the study populations’ subjective, perceived meanings, interpretations, and behaviors [31]. As Agar (1996) explained, qualitative research ‘humanizes stereotypes’ like SUD patients [32].

This study was part of the Pillars & Plants: Prevention, Support & Empowerment for Youth (PAPSE) project, which aimed to establish a chronic disease self-management clinical and e-therapy platform in a clinical trial and offer support group activities for adolescents and young people recovering from DSA. Africa University Clinical Research Centre, United Methodist Church Youth Pastors, and the Pastoral Care and Counselling Services provided the project support. The project was launched in 2019 and initially recruited and offered support to university students recovering from DSA. In 2019, young people recovering from DSA from the United Methodist Church circuits were recruited for weekly support group discussions with church youth pastors and mental health experts. The first cohort conducted face-to-face group sessions; however, the virtual platform was adopted for this second cohort due to COVID-19 pandemic restrictions. All participants were provided with data support to enable them to attend the weekly support sessions. The interviews of this study were conducted using young people recovering from DSA who had participated in the program’s second cohort (2020–2021).

### Study setting

The city of Mutare is in Manicaland Province, which borders Zimbabwe and Mozambique. After a long diamond rush discovered in 2006 in Chiadzwa communal areas near Mutare, many unemployed youths from all over the country and neighboring countries flocked to the city seeking to exploit the newly discovered economic opportunities. The city became a hub of illicit business, which attracted an influx of cheap alcohol and spirits from Mozambique.

According to the 2015 Zimbabwe Demographic Health Survey, 86% of Zimbabweans were Christians [33]. A local study revealed that about 75% of the Zimbabwean population consult spiritual and biomedical care providers for mental health [34]. Regarding mental health matters, some people shun the formal health system for being culturally insensitive [35].

### Study participants and recruitment

For the purpose of this study, the term youth was defined as any individual aged between 16 and 25 years of age. The study focused on a diverse group of church-going youths between 16 and 25 years recruited from the PAPSE DSA treatment program for young people recovering and DSA. The program recruited these young people from 28 United Methodist Church circuits in Mutare City to be part of support groups designed to offer them psychosocial support during their recovery. Eligible participants were youths in the support groups, and youth pastors who represented the church were recruited as key informants.

Invitations to participate in the study were sent to participants via the two WhatsApp groups, consisting of twenty DSA recovery program support group participants. Eighteen young people and three youth pastors responded to the invitation, and these were purposively selected for the interviews. The informed consent forms for those under 18 were signed by their parent/guardian. All the DSA recovery participants were black Africans, and 12 were males. Of the three-youth pastor interviewed, two were male, and their age range was 26 to 35. The key informants were purposively recruited from the pool of youth pastors who were actively involved in the weekly support group discussions. These key informants were interviewed on barriers and facilitators, as well as the role of the church in accessing psychosocial support among young people recovering from DSA.

### Data collection

The study participants received an informed consent form which they signed and returned to the researcher coordinator either in person or as a scanned document. The researcher then called the study participants to schedule the interview date and time. In-depth interviews were conducted by FFM and CM using semi-structured questionnaires for the youths recovering from DSA and the church pastors. Both interviewers were Zimbabwean citizens who understood all the languages used by the interviewees. Data collection was the first point of contact between the data collectors and study participants. Although the interviews were conducted in English, participants were encouraged to express themselves in the local language-Shona freely. The interviews were conducted using recorded telephone calls, and the files were saved on the cloud soon after the interview. A password-protected recording device was used as a backup, while phones were placed on ‘do not disturb mode during the interviews.

The researchers developed the questionnaires guided by variables associated with access to psychosocial support emerging from similar previous studies. The data collection tool was categorized according to the characteristics influencing psychosocial support for DSA recovery. First, participants’ sociodemographic characteristics (age, sex, marital status, and occupation) and experiences with substance abuse (duration of abuse, turning point, and the decision to seek help). Second, the barriers and facilitators for access to psychosocial support in the church context (disclosure; confidentiality; support from church leadership; stigma from church members, family, and peers) and the role of the church in DSA recovery (existence of psychosocial support structures, discussions around DSA among youths). Lastly, questions were asked regarding possible strategies to promote access to psychosocial support. The questionnaires were pretested using participants from the Africa University campus church circuit. The authors monitored the interviews for the saturation of themes. After interviewing the eighteen young people recovering from DSA and three youth pastors, the authors agreed that data saturation had been achieved, taking into consideration participant diversity and interview quality.

### Data analysis

The study followed the stages for the analyses in phenomenology. Data from the recorded interviews were transcribed verbatim for analysis by FFM and CM. The researchers translated the interview scripts into English, and nonverbal cues, e.g., sighs, pauses, and laughter, were included in the data transcription on a Microsoft Word document.

To improve accuracy in the interpretation of the interview transcripts, three research team members participated in the transcription of all the interview scripts. The research coordinator, FFM, led the analysis and had regular debriefing meetings with CM and FMM. An iterative qualitative research approach was applied to all the transcripts, which were read through multiple times to identify themes emanating from the interviews. Thematic network analysis of the qualitative data was conducted using techniques proposed by Attride-Stirling (2001) [36]. To facilitate decision-making and to problem-solve in an intelligible form while disentangling arguments, a thematic network enables the extraction of i) basic themes (lower-order premises in text), organizing themes (basic themes grouped), and global themes (overall themes capturing the principal metaphors in the text [36]. The thematic network used the hermeneutic approach to break up the text to find the embedded explicit rationalizations and implicit significance.

### Trustworthiness of the study

To enhance the trustworthiness of the study, all researchers underwent a mandatory Good Clinical Practice on Social and Behavioral Research Best Practices for Clinical Research training two weeks before data collection. The questionnaires were pretested using four young people and two youth leaders, and modifications were made before the interviews. Upholding data integrity and treatment fidelity was done by developing standard operating procedures for the data collection activities. To track fidelity and minimize protocol deviations, debriefing meetings were conducted on the morning of every day of data collection. These meetings were held throughout data collection and analysis. Discussions were used to resolve disagreements, and this was conducted until all parties involved reached a consensus. The researchers discussed with third parties who organized the PAPSE program and were responsible for the day-to-day running of the program to obtain feedback on the study results. We used quotes as a way of providing a vivid illustration of the participants’ responses on the questions asked. The Consolidated Criteria for Reporting Qualitative Research (COREQ) guidelines for reporting on qualitative research were used in reporting the study findings.

### Ethical considerations

The authors assert that all procedures contributing to this work comply with the ethical standards of the relevant national and institutional committees on human experimentation and

with the Helsinki Declaration of 1975, as revised in 2008. All procedures involving human subjects/patients were approved by the Africa University Research Ethics Committee (Approval number: AUREC 2460/22). Written informed consent was obtained from all participants, and permission to conduct the study was obtained from the Director of the Africa University Clinical Research Centre. For possible psychological adverse events during and after the interviews, the participants were referred to university psychotherapists who were available for free counseling via phone or email 24/7.

## Results

Table 1 displays the sociodemographic characteristics of the young people recovering from DSA who participated in this study. The primary outcome of interest, which was access to psychosocial support, was the global theme. The organizing themes were facilitators and barriers and the church’s role in accessing psychosocial support among young people recovering from DSA. The basic themes around facilitating factors were acceptance that DSA was a health problem that requires treatment, trust in the confidante, support from peers and parents, and a well-organized support program. The basic themes around barriers were having negative role models, radical religious beliefs that castigated drug-abusing youths, denial of one’s addiction problem, and societal stigma and discrimination of illicit drugs. The church may improve access to psychological support through rehabilitative programs and offer counseling services to young who have committed to quitting drugs.

**Table 1:** Characteristics of young people recovering from DSA who participated in this study

Variable	Characteristics	Frequency (n)	Percentage (%)
Sex	Male	11	61
	Female	7	39
Vocation	Formally employed		11
	Self-employed	2	11
	Not employed	9	50
	Student	5	28
Mean age (years)	21 ± 1.9		
Duration of DSA (years)		3	17
	2–4	8	44
	5–7	5	28
	8	2	11

The initial response reflecting on the study participants’ experiences revealed that substance use began during their years in school. The obstinate use of illicit drugs and substances led to dependance and SUDs. They talked about how they regret missing life opportunities because of substance abuse.

*“I started using drugs in high school. I was 15, and the senior boys I hung out with were doing it, and it looked cool. I failed to stop after that and dropped out of school. I am 24 now and have no hope for a future unless I stop this and accept that I need help.” (24, Male).*

Seven participants reflected on how they lost connection with family and friends. The subsequent loneliness and isolation intensified their drug-abusing habit.

### Facilitators

#### Individual acceptance

The study participants reported that acknowledgment of one’s drug addiction problem was integral to the recovery process. Understanding that using drugs was a perilous path that conflicts with Christian values and that there is a better and sober life led them to seek psychosocial support.

*“It was only when I accepted my drinking problem and that it had gotten out of control that I realized that I needed help.“ (21, Female). “I think I had a revelation. I started feeling empty, lonely… I needed God.” (22, Male).*

After realizing their mistakes, they noted how bad the situation had deteriorated and hence were willing to commit to new beginnings; as one participant described:

*“Self-reflection and being honest with myself is the only thing that made me seek for help. I thought I was headed for danger, but the truth was that I was already in danger.” (18, Male).*

Some participants had to witness the consequences of DSA for them to decide to quit.

*“I saw a dirty man lying face down in a roadside ditch. He looked dead. That was an awakening moment for me. I figured myself in his shoes, and I knew I had to stop!“ (20, Male).*

#### Confidentiality

The availability of someone the participant would trust emerged as a critical element in disclosing one’s drug addiction and ultimately seeking support. As one participant described: *“The bond I once had with one of the elders’ wives allowed me to be open up to her. Surprisingly, she did not judge me, and this took a huge load off my shoulders.” (22, Female).* The young people also felt that access to support from outside the church prompted them to seek counseling.

*“You can’t trust these ‘holy church youths’* (laughs); *they gossip a lot. I was so relieved when I talked to a church pastor from our church who was one of the counselors for the PAPSE Program. She was such a good listener, I trusted her, and I am glad she was good at keeping secrets.” (20, Female).*

#### Availability of an organized support program

Given the sensitivity of drug abuse issues in the community and the associated stereotypes, the interviewees wanted assurance of confidentiality. This was likely going to be possible if the support group was organized. One of the leaders stated:

*“We barely have efficient structures. It feels like we fail the youth who need us focusing on other issues. …we tend to neglect them.” (Male Pastor).*

One of the youths supported this:

*“Church pastors speak against substance use and encourage abstinence, but formal structures to support don’t exist. I believe drug recovery counseling for the church could be more effective if the pastors adapt the organized approach introduced by the PAPSE program.” (22, Female).*

#### Peer and parental support

Peer pressure was identified as the leading cause of drug uptake by the young people who participated in this study. However, peer support had a pivotal role in recovery. Young people can openly counsel each other, and peers who understand the dangers posed by DSA can be the compass to seeking help.

*“It was because of my friend that I felt I should seek help from professionals and God because I knew he had not forsaken me.” (21, Female).*

Despite having all their needs provided for at home, some young people still fall for DSA. The family’s involvement in the decision to seek support lightened the burden of solitude.

*“I had a very supportive family; they loved me and gave me attention and affection. I was the one who got lost and started doing drugs with the other children in my hood. I kept justifying my* (drug) *habit, but I am glad my parents never gave up on me until I had that turning point. I started attending church again…I still feel like a prodigal son from the Bible.” (25, Male)*.

### Barriers

#### Stigma and social rejection

When asked about barriers to seeking psychosocial support, the most central obstruction mentioned by the study participants was the stigma associated with DSA. The notion that some ‘sanctified’ youths who abstained from drugs were unwilling to associate with them made it difficult for the affected youths to seek support from within the church (from ten respondents).

*“They portrayed perfection in my eyes as good youths who did no wrong, and I felt they would not accept me and would judge me harshly.” (20, Female).*

The feeling that they were being discriminated against bore the assumption that they did not belong to the religious institution.

*“In their* (abstaining church youths) *eyes, I was that bad apple, an outcast with a bad influence on other youths. I did not see them accepting me as one of them.” (19, Female).*

#### Radical religious beliefs

As part of psychosocial counseling, some church elders prescribed seeking answers from the.

Bible, praying and fasting as a way of getting inspiration to quit drugs. Such generalized guidance did not align with the youths’ expected path to recovery.

*“I stopped seeking help from her* (church elder) *because she emphasized that I should study the Bible, believe it, and pray. That is a lot to read; she never pointed out where I should read from for the formula to succeed.“ (25, Male).*

Some relatives and the community may perceive DSA as a mystic malady caused by external forces like demons, witchcraft, or evil spirits, which causes indirect discrimination.

*“I heard people saying someone in my family had bewitched me, and they were making a fortune out of my misery… One day, I overheard my mom telling one of the church elders to pray for me so that the evil spirit in me would go away…I understand she wanted to help, but I was infuriated by her walking around saying these things about me.” (18, Male).*

Another major barrier to seeking psychosocial support was a stigma related to a lack of mental health literacy. Some appointed youth leaders were perceived as too old to understand the mental health problems faced by the current generation of youths.

*“The age gap* (between the young people and some youth pastors) *makes everything hard. They would not understand what exactly is going on… their main thrust and the ultimate solution would be praying for you to cast out demons when I need a compassionate ear.“ (24, Male).*

#### Denial

Some parents dissent from the news that their child abuses drugs, especially when the child.

goes to church. One of the youth pastors mentioned this:

*“No parent wants to believe that their child is doing drugs… that can be a sign of parenting failure.“ (Female Pastor).*

Parents or guardians always want the best for their children and tend to look away from the dark side of the child’s behavior thus missing an opportunity to help.

*“I told my aunt about my addiction problem, and she was adamant that we would pray and fast, and the problem would go away… It didn’t!“ (16, Male).*

#### Negative role models

One of the barriers to seeking psychosocial support was negative peer pressure and the associated temporary cushion it brings.

*“I started drinking with this group of boys I looked up to. They accepted me and gave me more attention than my parents did. I needed that honestly; unfortunately, the affection came with a terrible addiction.“ (18, Male).*

In some circumstances, the youth could not see the point of seeking psychosocial support because those who were supposed to help were also in a similar predicament.

*“Seeking help from youth leaders who are substance abusers is like a blind man leading another blind man.“ (20, Female).*

Seeking support can be difficult in situations where the family is also involved in substance abuse.

*“Since my mother died, my father has turned to intoxicating alcohol to drown his sorrows. I couldn’t rely on him for my recovery.“ (18, Male).*

### The role of the church

The participants’ narratives identified the church as a potential protective environment to provide counseling services for young people recovering from DSA. The church can implement this effectively through the various opportunities for prosocial activities against defiant behavior like substance use.

*“As leaders, we ought to identify the needs of the youths and exert our attention in that direction. We have to speak to all youths and not discriminate. We should create a safe and nurturing environment for those struggling with an open mind.“ (Female Pastor).*

Developing innovative ways of keeping the youths engaged in productive tasks to deter them from risky activities such as DSA.

*“As a church, we must meet the socioeconomic needs of the young people facing life challenges before seeking to restore their faith.“ (Male Pastor).*

When asked to elaborate on how these needs can be met, the pastor added:

“*We have groups such as choir, sports teams, and outings that are currently inactive. We should stimulate the youths to become active and join. Some problems need professional help… more than just prayer, and we should refer these to those trained to do the work.“*

As a source of Supreme Power through individual belief, the church can be a fortress that protects young people from DSA. One participant echoed this:

*“I knew my faith in God could help me sober up. I grew up in a Christian household and stopped attending church due to drugs. Restoring my faith in God would help me sustain my resolution to stop drugs.” (20, Female).*

One shared feeling reported as the church’s role was:

“*An affiliation with other progressive youths gave me solace… that dedication to serve the Lord has kept me from drugs.“ (17, Male).*

### Strategies to promote access to psychosocial support

Findings from the interviews revealed the need for increased awareness of current youth challenges. The campaigns should be able to reach out to youths within and outside the church since the church is a safety net for the vulnerable.

*“As Christians, we should have a welcoming attitude even to those who abuse substances and are not part of our church… interact with them in the community, listen to their side of the story and intervene on a case-by-case basis than judging from a distance or imposing our interventions on them.” (Male Pastor).*

To overcome idleness, the church should create opportunities for youths, especially those out of school.

*“The church should encourage and support youths to start projects through seed grants or promote sporting and entertainment activities to identify talent… some of the addiction is a result of unemployment… giving them* (youths) *some responsibility prevents indolence.” (24, Female).*

**Fig. 1:**
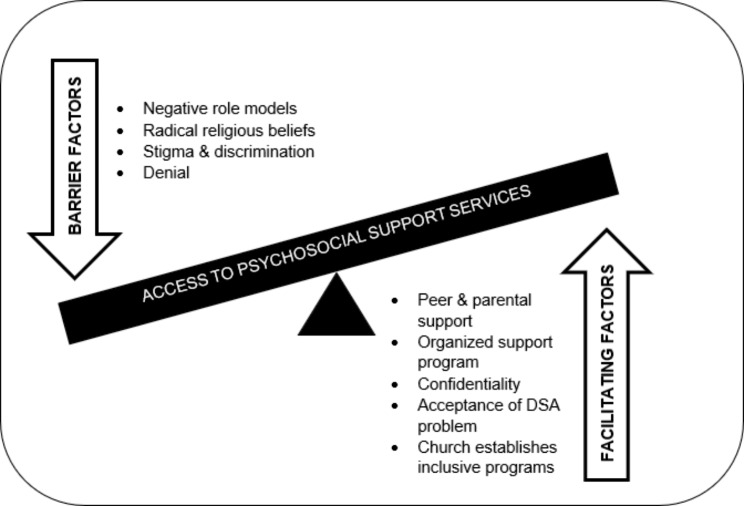
The thematic networks of access to psychosocial support for church-going young people recovering from DSA

Figure 1 shows the thematic networks for accessing psychosocial support among church-going youths.

## Discussion

Under the facilitating factors organizing theme, the researchers noted the following basic themes acceptance, confidentiality, peer and parental support, and organized support program.

Negative role models, radical religious beliefs, denial, stigma, and discrimination were identified under the barrier factors organizing theme. The church was seen as the arbitrator between the barriers and accessing psychosocial support (global theme) among church-going youths. The church ensures that young people get involved in projects and programs that avert idleness.

### Facilitators

Individual acceptance of the addiction problem facilitated access to treatment and support. Concerning mental health issues, acceptance is pivotal for coping and recovery [37]. Acceptance creates room for a positive mindset for behavior change associated with positive and clinically relevant outcomes [38, 39]. Without self-acceptance, the mental health problem will persist, and beneficial interventions will be less helpful [40]. The self-conscious recognition that one needs treatment should be emphasized while counseling young people recovering from DSA because it has a bearing on the retention of patients in treatment.

Confidentiality was one of the facilitators of accessing psychosocial support among the church-going youths who participated in this study. Trusting and confidential relationship issues are sensitive among young people’s engagement with mental health services providers [41]. Thus, facilities and individuals providing counseling services should be discreet and youth-friendly by offering open engagement, truthful responses to identified problems, and ensuring retention to care [42]. Training the youth pastors tasked with counseling duties in related ethical issues and providing a private environment for the counseling sessions is pivotal to attracting young people recovering from DSA in a church context. A similar study also echoed the need to train church leaders to handle SUD victims [43].

Peer and parental support were common facilitators to accessing counseling among the study participants. Openly disclosing one’s addiction challenges is facilitated by an established trusting relationship [41]. With support from peers and family members, the youth facing addiction challenges can build a buffer against social stigma and thus repel isolation and the associated anxieties [44]. Family and friends can be trustworthy, confident, and treatment buddies throughout recovery. Peer support groups encourage those experiencing SUD recovery to express their feelings, thoughts, and personal concerns in an environment that does not make them feel discriminated against.

The youths interviewed in this study applauded the development of the PAPSE program and how it incorporated the church leaders in an organized way that met their counseling needs. Organized and goal-directed programs motivate the youths to seek treatment because they provide systematic and explicit guidance [45]. A well-coordinated program reduces ambiguity and conflict, while treatment milestones can be measurable through mutually set targets. In contrast, the lack of professionalism and unplanned environments where therapists may fail to uphold ethical standards can deter young people from seeking mental health treatment [46]. Standardized structures devoted to psychosocial support of young people recovering from DSA in places of worship in Zimbabwe are limited and we recommend that the religious organizations institute these to improve access to support for this vulnerable population.

### Barriers

We noted that stigma and social rejection were significant barriers to accessing psychosocial support among young people recovering from DSA. This is consistent with other studies in which fear of rejection was a concern among individuals struggling with addiction which would be perceived as weak by their peers and significant others [47–49]. The internalized belief based on misconceptions about self significantly influences well-being, ultimately affecting access and utilization of health services [50]. While drug addiction is a source of stigma, the thought of social rejection after disclosing one’s problem can exacerbate mental health challenges. A transparent channel and non-judgmental approaches can encourage more young people to disclose, seek and access SUD treatment early.

Some church counselors emphasized reading the Bible, fasting, and prayer, a strategy noted as ineffective by the study participants. One of the effective strategies to heal mental health problems is genuine, empathetic listening [51]. Receptivity and good listening skills by the counselor allow the victim to freely express themself without feeling that they are being judged. These can be achieved using motivational interviewing techniques guided by four principles: resisting the righting reflex; understanding the patient’s motivations; listening with empathy; and empowering the patient [52]. The counselor should allow the patient to confide without rushing to prescribe solutions since individuals with addiction challenges often feel misunderstood. A counselor’s patience permits understanding the problem from the patient’s perspective and provides a specific solution.

Instances of negative role models were noted as a barrier to seeking support among church-going youths facing drug addiction challenges. Trusted primary sources of attachment, like family members, can be a source of a refugee during the recovery. However, the knowledge that the individual who should be part of the solution is also part of the problem can induce negative thinking and resentment. Parents who have SUDs are likely to have unhealthy family functioning styles and lower care scores [53]. Living or constantly associating with someone still using substances can trigger cravings that may lead to relapse [54]. Thus, to improve treatment outcomes, DSA counselors need to have an upstream mentality and understand the conditions in which their clients reside.

Similarly, church leadership for the youth and those who appoint addiction counselors should do so after a careful assessment that includes background checks. The treatment process can be impeded if the parents/guardian of the child is in denial. The parent often feels responsible when their child is a DSA leading to denial and experiences such as stress, self-accusations, anger, sadness, and pressure to assist their child to overcome the addiction problem [55, 56]. The counselor must understand the patients’ social support base to provide comprehensive therapy.

### Role of the church and strategies to promote access to psychosocial support

Religious organizations are critical in offering recovery support services, coping ability, and rehabilitation of DSA patients [51]. Youth involved in faith-based activities exhibited better resilience and the ability to cope without using drugs when compared to their counterparts [28]. Introducing social activities to reduce idleness or financial gains can positively impact the recovery program. Volunteering for charity work for congregational substance abuse recovery programs is associated with positive health outcomes [57]. Volunteerism help patients reconstruct their new identity towards recovery, and besides a good feeling of self-worthiness, helping others improve self-esteem and optimism while reducing depression and helplessness. Rendering service to the community in the substance treatment field can also sustain one’s mental health recovery and maximize multiple aspects of life functioning [58].

Young people expect to be financially independent, and the alarming unemployment rates in Zimbabwe can be a barrier to alcohol and DSA recovery, even with good counseling. This was also noted in a study by Stokes and colleagues (2018) [39]. Employment is deemed one of the critical indices of accessing psychosocial support among drug addicts, as work provides opportunities for socialization and healthy relationships with non-substance abusers [59]. Thus, strengthening commitment to recovery [60]. Recovery programs within and outside the church should forecast improving the employability and source training for the youth to prevent relapse. In addition, the church can also advocate for the recovering youths in a way that will improve a positive employer attitude. To succeed in its mandate, Christians must reflect on self-concept and reinforce the culture of love and respect.

### Strengths and limitations

This study brought novel knowledge of the challenges faced by church-going youths, and according to the authors’ knowledge, it is the first of its kind in Zimbabwe. However, our study had some limitations as it was conducted using young people from one Christian denomination.

Thus, the findings cannot be generalized to other churches and contexts. All the participants were self-selected from the youth involved in the PAPSE program leaving out those who had the addiction but had not enrolled for treatment. Using self-reports may be a source of bias as participants can give socially desirable responses. Therefore, a more robust quantitative study could be conducted to enhance reliability.

## Conclusions

Acceptance of one’s addiction problem is critical to initiate seeking psychosocial support. Confidentiality with disclosed information, support from trustworthy relationships, and the availability of a well-coordinated recovery program enable young people to seek support. An

organized church-mediated drug abuse support program has the potential for high-success treatment outcomes and retention. By providing innovative projects, inclusive programs, and social support, the church can bridge the psychosocial support gap between the support services and the barriers, mostly interactive relationship based. Formal training of church-based counselors or pastors can have profound treatment outcomes by introducing the ethical aspects of psychotherapy and reducing the preconceived social stigma associated with SUDs. To assist youth in prompt access to mental health support services, Christians willing to help must approach this sensitive age group with an altruistic lens.

## Data Availability

The datasets used and/or analyzed during the current study are available from the corresponding author on reasonable request.

## Declarations

### Authors’ information

The researchers hold the following credentials: FFM (BSc Psychology), PTM (BSN, MPH, Ph.D. Student), CM (M. Ed in Educational Psychology, BSc Psychology), and FMM (MPH, MD). During the study period, FFM was an undergraduate student, PTM was a Research Support Officer, CM was a senior lecturer, and FFM was the Director of the Africa University Clinical Research Center. All researchers were female except PTM. FFM and CM had one and ten years of experience in social science research, while PTM and FMM had seven and 13 years of experience in public health research, respectively.
